# Comparative evaluation of surface roughness and bacterial adhesion on two bioactive cements: an in-vitro study

**DOI:** 10.1186/s12903-024-05083-y

**Published:** 2024-10-24

**Authors:** Pallabi Dey, Baranya Shrikrishna Suprabha, Ethel Suman, Srikant Natarajan, Ramya Shenoy, Arathi Rao

**Affiliations:** 1 Department of Pediatric and Preventive Dentistry, Manipal College of Dental Sciences Mangalore, Manipal Academy of Higher Education, Manipal, Karnataka 576104 India; 2 Department of Microbiology, Kasturba Medical College Mangalore Manipal Academy of Higher Education, Manipal, Karnataka 576104 India; 3 Department of Oral Pathology and Microbiology Manipal College of Dental Sciences Mangalore, Manipal Academy of Higher Education, Manipal, Karnataka 576104 India; 4 Department of Public Health Dentistry Manipal College of Dental Sciences Mangalore, Manipal Academy of Higher Education, Manipal, Karnataka 576104 India

**Keywords:** Biofilm, Bacterial adhesion, *Streptococcus mutans*, Composite resins, Glass Ionomer cement

## Abstract

**Background:**

Dental restorative materials are recognized as artificial niches that facilitate the adherence and accumulation of oral microorganisms. To mitigate oral diseases and extend the lifespan of restorations, it is advantageous to use dental materials that exhibit low susceptibility to bacterial adhesion.

**Objective:**

To evaluate and compare bacterial adhesion on two bioactive restorative materials, a glass hybrid restorative, and an alkasite with a nanohybrid resin composite as a positive control. The secondary objectives were to compare the surface roughness (SR) of the materials and determine the correlation between the bacterial adhesion and the SR.

**Materials and methods:**

The samples consisted of 33 polished discs of each material: Group A: Tetric^®^ N-Ceram (nanohybrid resin composite), Group B: Equia Forte™ HT Fil (glass hybrid restorative) and Group C: Cention N^®^ (alkasite). *Streptococcus mutans* cultures were inoculated and after 24-hours of incubation, bacterial adhesion was measured by measuring optical density (OD) and number of colony forming units (CFUs). After 96-hours incubation, the bacterial cell count was determined using scanning electron microscopy (SEM). SR was assessed using surface profilometer.

**Results:**

Alkasite had significantly lower OD and CFUs (*p* < 0.001 and *p* = 0.015 respectively). According to the SEM analysis, the glass hybrid restorative had lower mean bacterial cell count with no significant difference between the groups. The nanohybrid composite had the smoothest surface that was significantly lower than the alkasite and glass hybrid restorative (*p* = 0.002). None of the groups demonstrated a correlation between bacterial adhesion and SR.

**Conclusion:**

Alkasite impedes bacterial adhesion better than the glass hybrid restorative and nanohybrid composite, while smoother surfaces are achieved with the nanohybrid composite.

## Introduction

Dental restorative materials are recognized as artificial niches that facilitate the adherence and accumulation of oral microorganisms. The dental plaque or biofilm on restorative material surfaces contain many bacteria that are involved in the demineralization process along the restorations margins which can lead to secondary caries [1]. Among the *Streptococcus* species that predominate the biofilms, *Streptococcus mutans (S. mutans)* can adhere to tooth and restorative material surfaces and hence are considered as the primary cause of secondary caries formation [2].

The ability of restorative materials to attract bacterial adhesion is affected by the surface characteristics of the material such as surface roughness, surface free energy, and chemical composition [3]. A well-polished smooth surface of the restoration prevents plaque accumulation and marginal secondary caries. It improves patient comfort and oral hygiene maintenance [4].

Among the various restorative materials, resin composites with nanofiller particles have been conventionally used due to their superior mechanical properties, translucency, surface smoothness, polishing ability, and gloss stability [5, 6]. Nanohybrid composites such as Tetric^®^ N-Ceram have both nano and micro sized filler particles that result in improved mechanical properties such as strength, fracture toughness and modulus of elasticity [5, 7]. However, resin composites continue to be susceptible to secondary caries due to marginal gaps [8].

Restorative materials that are bioactive form a bond between the tooth tissues and materials. They release ions in the presence of biological fluids such as saliva and induce the formation of apatite-like material leading to remineralization, which maintains marginal integrity and prevents secondary caries occurrence [8, 9].

Glass ionomer cements (GICs) are a group of materials with thermal expansion like that of dentin, minimal cytotoxicity and good biocompatibility [10]. The material undergoes slight hygroscopic expansion that results in marginal gap closure, reduced microleakage and hence recurrent caries [11]. These cements are bioactive because the fluoride released by the material inhibits demineralization and promotes remineralization of the adjacent tooth tissues [12]. GICs have undergone continual modifications in their compositions to improve their mechanical properties, polishability and aesthetic appearance. The GICs were reinforced with fillers to form high viscosity GICs which were further modified using glass hybrid technology and marketed as Equia Forte^®^ Fil, which contains highly reactive small glass particles and increased polyacrylic concentration. It contains strontium fluoroaluminosilicate glass and has superior mechanical properties than otherGICs [13]. The material releases more fluoride and is indicated for posterior stress-bearing restorations [14]. Recently, it was further modified to Equia Forte ^TM^ HT Fil that uses glass hybrid technology, reinforced with ultrafine and more reactive silicate particles to further improve the mechanical qualities, material flow, non-stick handling properties and translucency, thus mimicking the properties of resin composites [15].

Cention N^®^ is a more recent composite restorative material made of bulk-fill resin and alkasite fillers [16]. The flexural strength, fracture resistance, and bond strength of Cention N^®^ are similar to those of traditional composite resins [16, 17]. The material also shows lower polymerization shrinkage and microleakage in comparison to other restorative materials [18]. At low pH due to the acids produced by the bacteria, remineralization is promoted due to the release of hydroxyl, calcium, and fluoride ions by the alkasite [8]. Hence alkasite is an alternative restorative material to resin composites due to its bioactive properties.

Earlier studies comparing the bacterial adhesion and surface roughness of GICs with resin composites have produced inconsistent findings [19–21]. Recent systematic or narrative review on bacterial adherence to dental restorative materials have reported data on bacterial adhesion to resin composites and GICs, but information on bacterial adhesion to bioactive materials appears to be limited [21, 22]. Daabash et al. [23] studied the adhesion of *S. mutans* to ion releasing resin-based composites, Activa ^TM^ and Cention N^®^ in comparison with a conventional resin composite (Z350) and a resin-modified GIC (Fuji-II-LC). There was no statistically significant difference between the bacterial adhesion of Cention N^®^, Fuji-II-LC and Z350. However, due to ion releasing properties, Cention N^®^ and Fuji-II-LC groups had more dead bacterial cells. In another study by Bohnic et al. [20] bacterial adhesion of nanohybrid (Tetric Evo Ceram) and micro hybrid resin (TE Econom) composites were compared with conventional (Fuji IX) and hybrid GICs (Equia Forte Fil). It was found that both conventional and hybrid GICs had significantly lower bacterial adhesion, which was attributed to lower surface roughness. The literature search did not reveal data on the comparative bacterial adhesion to bioactive restorative materials like Equia Forte™ HT Fil, a recently available improved version of glass hybrid restorative material and Cention N^®^, an alkasite.

Thus, we aimed to assess and compare the bacterial adhesion to two bioactive restorative materials, bulk fill glass hybrid restorative (Equia Forte ^TM^ HT Fil) and alkasite (Cention N^®^), with a conventional restorative material such as a nanohybrid resin composite (Tetric^®^ N-Ceram) serving as a positive control. The secondary objectives were to compare the surface roughness and to assess the relationship between the bacterial adhesion and surface roughness characteristics of the bioactive restorative materials. This study was conducted based on the null hypothesis that there is no difference in bacterial adhesion and surface roughness between the bulk fill glass hybrid restorative, alkasite, and nanohybrid composites.

## Materials and methods

### Study design and sample size

For this in vitro study, the sample size was calculated assuming an effect size of 20%, 50% relative precision, 95% confidence interval, and 80% power, using G Power 3.1.2 software (Heinrich Heine University Düsseldorf, North Rhine-Westphalia, Germany). The sample size arrived at was 11 in each group.

### Sample grouping

The samples consisted of discs made of the three restorative materials, constituting the three groups: Group A: Tetric^®^ N-Ceram (TNC) (positive control), Group B: Equia Forte ^TM^ HT Fil (EF) and Group C: Cention N^®^ (CN). The composition of the materials used in each group is provided in Table 1.

**Table 1:** Composition of the restorative materials and polishing system used in the study

Material	Type	Manufacturer	Composition
Equia Forte™ HT Fil [12]	Glass Hybrid Bulk Fill Restorative	GC Corporation, Tokyo, Japan. Lot No.2,201,261	**Powder**: 95% strontium– fluoroaluminosilicate glass, 5% polyacrylic acid **Liquid**: 40% aqueous polyacrylic acid.
Cention N^®^ [21]	Alkasite Resin Composite	Ivoclar Vivadent AG, Schaan, Liechtenstein. Lot No. Z039ZR	**Powder**: calcium fluorosilicate glass, barium glass, calcium-barium-aluminum fluoro-silicate glass, iso-fillers (Tetric^®^ N‑Ceram technology), ytterbium trifluoride, initiators, and pigments **Liquid**: dimethacrylates, initiators, stabilizers, additives, and mint flavor.
Tetric^®^ N-Ceram [24]	Nanohybrid Resin Composite	Ivoclar Vivadent, Schaan, Liechtenstein Lot No. Z04WW4	**Resin**: Bis-GMA, Bis-EMA and urethane dimethacrylate monomer (UDMA), involving advanced composite-filler technology, patented light initiator Ivocerin^®^ **Filler**: Barium aluminum silicate glass with two different mean particle sizes, Filler content approximately 61% (volume) and 17% polymer fillers or “Isofiller”
Sof-Lex™ Spiral Diamond Polishing System [25]	Finishing Polishing System with Aluminum oxide and diamond impregnated discs	3 M ESPE, St. Paul, MN, USA. Lot No. NF14746	Thermoplastic elastomer impregnated with aluminium oxide particles and diamond abrasive particles.

### Specimen preparation

Using a plastic filling instrument, thirty-three samples of each group were prepared by compacting the materials into round shaped metal moulds (8 × 2 mm^2^). All the specimens were prepared following the instructions provided by the manufacturer. Briefly the details of material mixing, and manipulation are as follows:

EF was mixed using a capsule mixer for ten seconds, (Telematic and Biomedical Services Pvt Ltd, Mumbai, India) after which, the capsule was placed in a metal GC Capsule Applier (GC, Tokyo, Japan), primed, and poured into the moulds in less than ten seconds. Light-curing was done for 20 s after the excess material was removed [24].

For CN, the powder and liquid were mixed on mixing pad at a powder/liquid ratio of 4.6:1. The mixing process lasted 45 to 60 s and light curing lasted for 40 s [25, 26]. TNC was applied using plastic filling instrument and light-cured for 20 s [27].

An explorer was used to remove the excess restorative material. The mould was lubricated using petroleum jelly (Vaseline, India) before placing the restorative materials. To create a smooth surface, the mould was sandwiched between two transparent mylar strip (Nexus, Medodent, India) and glass slides that were one millimetre thick, employing finger pressure, taking care to avoid the inclusion of air bubbles or folds into the restorative materials. Light curing was performed at > 500mW/cm^2^ light intensity, to form discs. The curing light (Bluedent LED Smart, BG Light Ltd, Plovdiv, Bulgaria) intensity was measured using a calibrated radiometer (Demetron 100, Demetron Research Corp, Danbury, CT, USA) for every fifth sample. Following removal from the moulds, the specimens underwent a two-step finishing and polishing process using Sof-Lex™ spiral aluminium oxide and diamond polishing discs (3 M ESPE, St. Paul, MN, USA) following the manufacturer’s guidelines [28].

The exclusion criteria for the prepared specimens were inaccurate dimensions, gaps, fractures, or chipped edges. The specimens were immersed in deionized water for a full day prior to every test. One single operator handled all the steps of the specimen preparation, finishing, and polishing process.

### Assessment of bacterial adhesion

#### Preparation of streptococcus mutans cultures

5% sheep blood agar (Hi-Media Media Laboratories Pvt Ltd Mumbai, India) was used as the medium for the *S. mutans* strain (ATCC 25175, Hi-Media Media Laboratories Pvt Ltd Mumbai, India), which was cultured at 37^0^C for 48 h in a CO_2_ incubator (Nuaire, Plymouth, USA).

Colonies of *S. mutans* were inoculated into 10 ml of brain heart infusion (BHI) broth (Hi-Media Media Laboratories Pvt Ltd, Mumbai, India) supplemented with 5% glucose, and incubated at 37 °C for 24 h, to encourage further bacterial growth. To collect the bacterial cells, the cultures were then centrifuged (REMI R-8 C BL, Mlabs, Delhi, India) at 4000 rpm for 15 min. After discarding the supernatant, the bacterial residue was washed twice with sterile phosphate-buffered saline (Hi-Media Media Laboratories Pvt Ltd, Mumbai, India). The density of the bacterial cells was then standardized to 0.5 McFarland, equivalent to 1.5 × 10^8^ CFU/ml.

#### Optical density (OD) and colony forming units (CFU) measurement

A total of 22 discs were randomly selected from each group and autoclaved at 121 °C for 20 min before the bacterial adhesion analysis. The adhesion analysis was done in duplicate. All the samples were placed in a 24-well flat-bottomed tissue culture well. Two discs of each material were placed in each well, one for analyzing bacterial adhesion by measuring the OD and CFU, and the other for measuring bacterial biofilm formation using a scanning electron microscope (SEM). After inoculating the specimen-containing wells with 200 µl of *S. mutans* culture (1.5 × 10^8^CFU/ml), 1800 µl of BHI broth containing 5% glucose was added and incubated in a carbon dioxide incubator for 24 h at 37 °C. After the incubation, one sample from each well was twice dip-washed with phosphate buffered saline to remove any unattached bacteria, placed in a sterile Eppendorf tube (Eppendorf India, Chennai, India) with 1 ml of phosphate buffered saline, and vortexed [REMI CM101, Mlabs, Delhi, India) at 2500 rpm for 60 s to collect the adhering bacteria. 200 µl of the vortexed samples were then placed in a 96-well microtiter plate and the OD was measured spectrophotometrically (ELISA Reader ELX-800, Biotek, Vermont, USA) at 650 nm (OD_650_). Colony counting by surface plating method was also performed on BHI agar with 5% glucose using logarithmic dilutions of the vortexed sample followed by incubation for 48 h at 37 °C. The number of colonies were expressed as CFU/ml.

##### Bacterial count measurement with Scanning Electron Microscopy (SEM)

After 96 h of incubation in BHI broth supplemented with 5% glucose, six samples were randomly selected from each group, from the 11 duplicate samples remaining in the tissue culture wells, for SEM analysis. SEM (EVO MA18, Carl Zeiss AG, Oberkochen, Germany) was used to observe bacterial biofilm formation.

Biofilm development was verified by gram-staining the scrapings from one disc in each of the groups. After gently washing the samples in phosphate-buffered saline, 2.5% glutaraldehyde was used for fixation and then the samples were dehydrated by washing with increasing concentrations of ethanol for 15 min at a time: 50%, 70%, 85%, 90%, and 100%. After being dehydrated, the samples were carefully mounted on an acrylic stub. Next, the samples were inspected at 10,000× magnification after being sputter coated with a 6 nm layer of gold. The images were analyzed both qualitatively and quantitatively. At 10,000× magnification, the number of *S mutans* cells that adhered to the material surface was counted using the ImageJ software (version 1.50b, National Institutes of Health, Bethesda, USA).

Using the software, 100 µm^2^ grids were created to standardize the image size. Using the measurement tool, the bacterial cells were counted in the two randomly selected neighbouring grids of each image and represented as the number of cells per 200 µm^2^ of the specimen surface. The counts were obtained over five areas of each specimen, and the mean was calculated.

#### Surface roughness

**Fig. 1:**
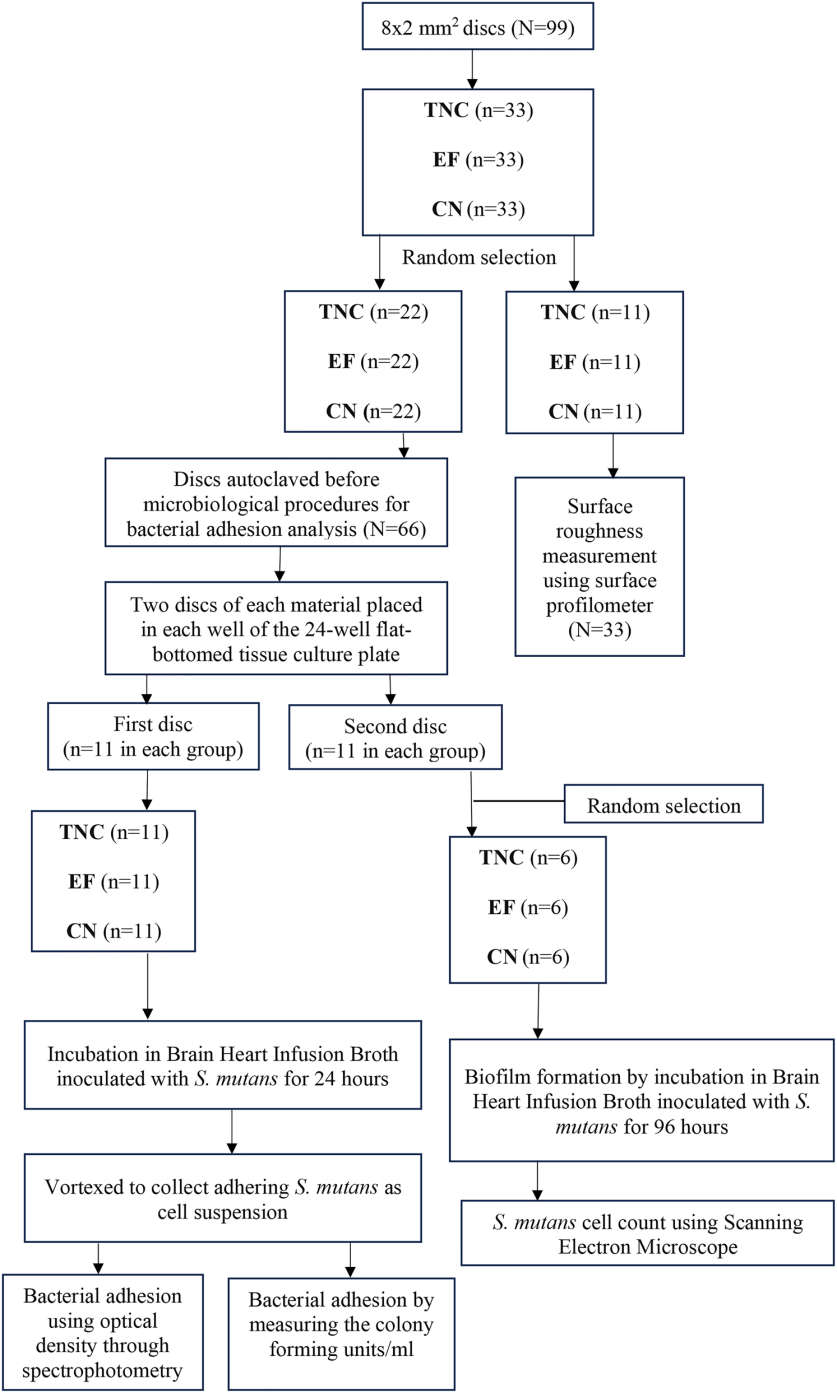
Flow chart representing the flow of the specimens for bacterial adhesion measurement. TNC-Tetric^®^ N-Ceram (Nanohybrid resin composite-positive control); EF-Equia Forte ^TM^ HT Fil (Glass hybrid restorative material) and CN-Cention N^®^ (Alkasite)

The surface roughness (SR) of the remaining 11 samples from each group was assessed using a surface profilometer (Dektak XT^®^, Bruker, MA, USA) with a 12.5 mm stylus diameter, a force of 0.03 mN, at a distance measurement of 4 mm and an amplitude height of 2.5 mm. Three profiles were collected from the centre, right, and left sections of each specimen using a constant measurement speed of 40 mm/second. Before every new measurement session, the profilometer was calibrated against a standard. For each sample, the SR value (Ra) was calculated, and the mean Ra values were obtained in µm. During the determination of all the study parameters, the operator was blinded to the material type of the disc. The flow of the study specimens is represented in Fig. 1.

### Statistical analysis

Data was analyzed using IBM SPSS software, version 29. Descriptive statistics were calculated, and normality was assessed using the Shapiro-Wilk test. Non-parametric tests were used if the test was significant. The difference between the groups for OD values, CFU counts, and Ra values were analyzed using the Kruskal-Wallis test. Further pairwise comparisons were done with Mann-Whitney U tests. One-way ANOVA was done for SEM analysis data. The significance level was at *p* ≤ 0.05 for all the tests except the Mann-Whitney U test, where the type-I error due to multiple intergroup comparisons was controlled by Bonferroni correction (*p* ≤ 0.016 significant).

## Results

The OD_650_ values of the alkasite (CN) group were lower than those of the nanohybrid resin composite (TNC) and glass hybrid restorative groups (EF), which had the highest values. Overall, the difference in the optical density values was between the groups was statistically significant (*p* < 0.001) (Table 2). TNC differed significantly from the CN and EF groups (*p* < 0.001), and a statistically insignificant difference was observed between the EF and the CN groups (*p* > 0.016, not significant according to Bonferroni correction) (Table 3).

**Table 2:** Intergroup comparison of bacterial adhesion and surface roughness (Ra)

Variable	Group	*N*	Mean ± SD	Median (IQR)	H value	*p* value
OD_650_	TNC	11	0.21 ± 0.07	0.21(0.08)	23.73	< 0.001*
	EF	11	0.03 ± 0.01	0.02(0.01)		
	CN	11	0.02 ± 0.01	0.01(0.01)		
Log CFU/ml	TNC	11	6.45 ± 0.13	6.45(0.23)	8.36	0.015*
	EF	11	6.50 ± 0.16	6.48(0.35)		
	CN	11	6.29 ± 0.15	6.28(0.30)		
Ra (µm)	TNC	11	0.08 ± 0.01	0.09(0.03)	12.79	0.002*
	EF	11	0.16 ± 0.04	0.15(0.07)		
	CN	11	0.15 ± 0.06	0.20(0.12)		

*p ≤ 0.05 = Significant; SD = standard deviation; IQR = Interquartile range; OD_650_ = optical density at 650 nm; Log CFU/ml = Logarithm of number of colony-forming units per milliliter; TNC-Tetric^®^ N-Ceram (Nanohybrid resin composite-positive control); EF-Equia Forte ^TM^ HT Fil (Glass hybrid restorative material) and CN-Cention N^®^ (Alkasite)

**Table 3:** Multiple pairwise between group comparisons of bacterial adhesion and surface roughness (Ra)

Variable	Pairwise comparison of Groups	Mann-Whitney U	*p* value
OD_650_	TNC vs. EF	0.01	< 0.001*
	TNC vs. CN	0.01	< 0.001*
	EF vs. CN	30.50	0.039
Log CFU/ml	TNC vs. EF	51.50	0.554
	TNC vs. CN	25.50	0.021
	EF vs. CN	21.00	0.009*
Ra (µm)	TNC vs. EF	4.50	< 0.001*
	TNC vs. CN	24.50	0.017
	EF vs. CN	59.50	0.946

*p ≤ 0.016 = Significant [Bonferroni correction]; OD_650_ = optical density at 650 nm; Log CFU/ml = Logarithm of number of colony-forming units per milliliter; TNC-Tetric^®^ N-Ceram (Nanohybrid resin composite-positive control); EF-Equia Forte ^TM^ HT Fil (Glass hybrid restorative material) and CN- Cention N^®^ (Alkasite)

The Log CFU values of the CN group were lower than those of the TNC and the EF, which had the highest values. Overall, the difference in the Log CFU values was statistically significant between the groups (*p* = 0.015) (Table 2). Intergroup comparison of the EF and CN group using the Mann-Whitney U test showed significant results (*p* = 0.009), with no significant p values for the intergroup comparisons of the TNC with the EF and CN (*p* > 0.016, not significant according to Bonferroni correction) (Table 3).

**Fig. 2:**
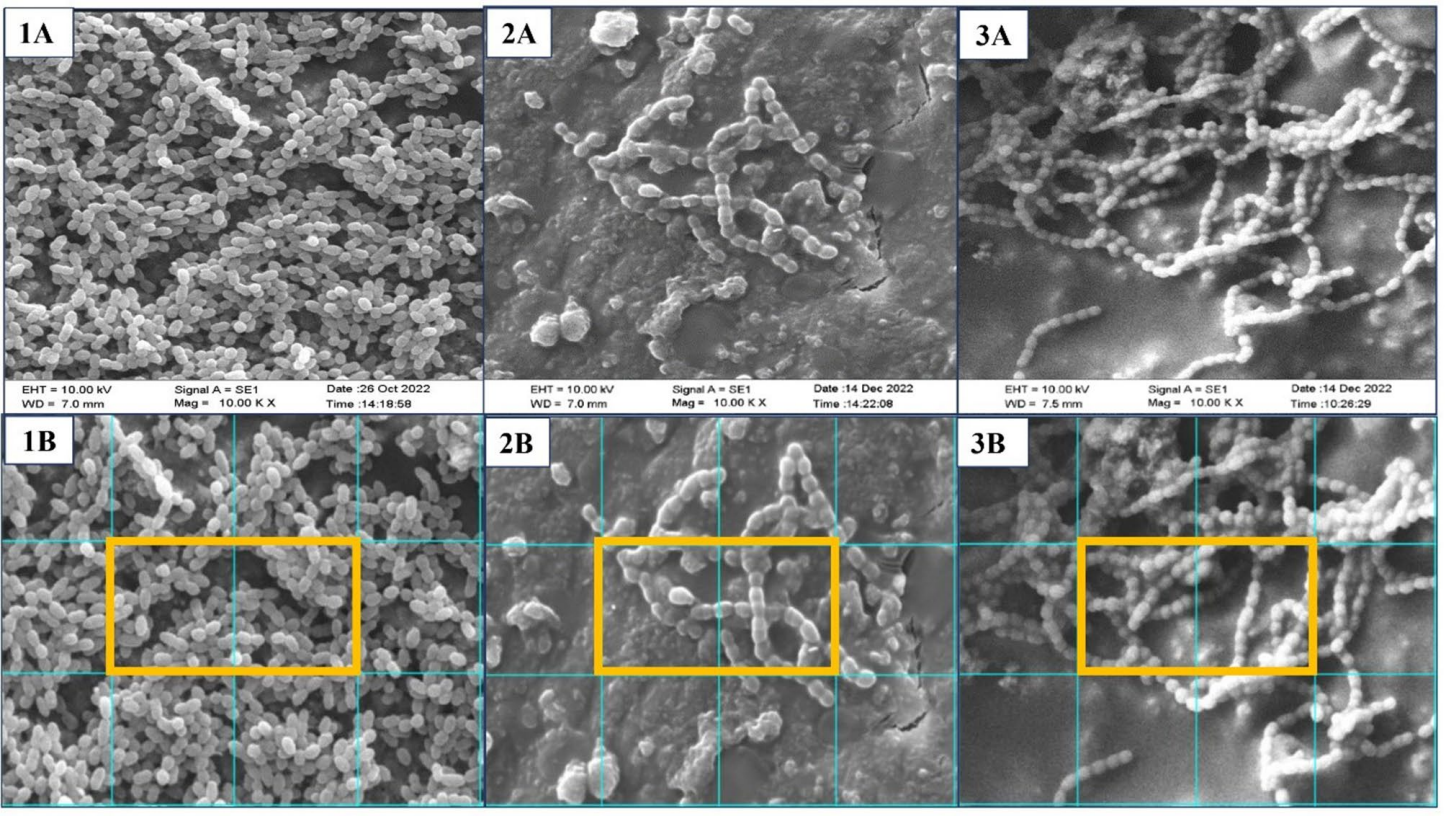
SEM image at 10000x magnification of the three groups: *S. mutans* cell counting was performed using grids by ImageJ software in three groups. The yellow highlighted area shows adjacent grids and represents cells per 200 µm^2^; 1(**A**) and 1(**B**): TNC [Positive control]; 2(**A**) and 2(**B**): EF; 3(**A**) and 3(**B**): CN

The SEM images of all specimens from the experimental groups at 10,000×magnification (Fig. 2) revealed biofilm formation by *S mutans*, visible as cocci chain formation of the bacteria. The cocci chains in the biofilms of the TNC and the CN group specimens were tighter and denser than the EF group. In the quantitative SEM analysis for the bacterial cell count/200 µm^2^, the area covered by the biofilm was 100% for the TNC, and 64% and 28% for the CN and EF groups respectively. The TNC (64.24 ± 31.59) and CN (51.98 ± 38.79) had greater mean bacterial cell counts per 200 µm^2^ than the EF (38.67 ± 22.36). Overall, the difference between the groups was not statistically significant (F = 0.981, *p* = 0.398).

In the SR measurement (Ra) obtained in µm, the TNC showed the lowest value, followed by the CN and EF groups. In terms of surface roughness, there was a statistically significant difference between the groups (Table 2). Overall, the surface roughness values ranged from 0.06 to 0.2 μm. The results of the intergroup comparison between the EF and TNC utilising the Mann-Whitney U test were significant (*p* < 0.001), with no significant differences between the CN and EF groups, or between the CN and TNC groups, after the Bonferroni correction (*p* > 0.016, not significant according to Bonferroni correction) (Table 3).

Non-parametric correlation (Spearman’s rho) of bacterial adhesion measured using OD_650_, and CFU parameters showed no significant correlation with SR for any of the groups (Table 4).

**Table 4:** Correlation of bacterial adhesion with surface roughness

Variable	Group	*r*_s_ value	*p* value
OD_650_	TNC	0.179	0.598
	EF	0.542	0.085
	CN	-0.186	0.604
Log CFU/ml	TNC	0.180	0.596
	EF	0.337	0.311
	CN	-0.280	0.559

OD_650_ = optical density at 650 nm; Log CFU/ml = Logarithm of number of colony-forming units per milliliter; r_s_= Spearman’s rho; TNC-Tetric^®^ N-Ceram (Nanohybrid resin composite-positive control); EF-Equia Forte™ HT Fil (Glass hybrid restorative material) and CN- Cention N^®^ (Alkasite)

## Discussion

Equia Forte™ HT Fil and Cention N^®^ are newer bioactive restorative materials with promising mechanical properties comparable to conventional nanohybrid resin composite, in addition to their bioactive properties [14, 16]. The findings of this study demonstrated that Equia Forte™ HT Fil and Cention N^®^ had lower bacterial adhesion but greater SR than did Tetric N^®^ Ceram that served as positive control. No correlation was detected between bacterial adhesion and the SR for any of the materials evaluated. Based on the study results, the null hypothesis that there is no difference between the restorative materials in terms of bacterial adhesion and surface roughness was rejected. However, in accordance with SEM results, we failed to reject the null hypothesis for bacterial biofilm formation. The physicochemical characteristics of both bacterial and material surfaces influence bacterial adhesion processes [3]. The hydrophobicity and surface energy of the material surface, the growth medium, and the bacterial strain are the factors that impact how bacteria interact with the substrate. *S. mutans*, has a high surface energy, is hydrophobic and shows high adhesion to hydrophobic materials such as resin composites and GICs [2, 3]. Higher contact angles which are a measure of hydrophobicity are known to increase bacterial adhesion. Materials with contact angles greater than 62^°^ are known to be hydrophobic. Evidence from the literature shows that the contact angles of the three materials included in this study are greater than the above threshold which renders them hydrophobic [2, 23]. The polishing procedure enhances the roughness and surface free energy further contributing to bacterial adhesion, thus all the materials in the study showed bacterial adhesion and biofilm formation [19].

Cention^®^ N has the hydrophilic monomer PEG-400DMA and *S. mutans* has a weak affinity for hydrophilic surfaces [2, 25], which may be the reason for the lower *S. mutans* adhesion observed in this study. The glass hybrid material is more hydrophobic with a contact angle of 97^°^and thus has a potential for greater bacterial adhesion [20]. The nanohybrid resin composite also has been shown to have high contact angles (around 80°) for water, rendering the material hydrophobic, thus making it susceptible to *S. mutans* adhesion. The hydrophobicity of the nanohybrid resin composite further increases due to polishing, which removes the organic matrix, and exposes the charged, hydrophobic filler particles [29, 30].

Material characteristics such as ion release and SR also influence the extent of bacterial adhesion [23]. Bioactive restorative materials release ions that have antibacterial activity and hence affect the bacterial adhesion [31]. The ability of GICs to release ions like fluoride and aluminium is known to inhibit the biofilm formation of *S. mutans.* Low fluoride concentrations can inhibit bacterial enzymes such as ATPase and hamper the acid production by bacteria [32]. Glass hybrid restorative materials also inhibit biofilm formation due to the release of strontium ions that potentiate the antibacterial effect of fluoride [33]. The glass hybrid restorative releases more fluoride than other GICs [14]. Although the glass hybrid restorative had a high degree of SR that encourages bacterial adherence, the burst release of fluoride and strontium ions for up to 4 days can have an inhibitory influence on biofilm development [33], as shown by the OD or CFU values of the glass hybrid restorative material measured at 24 h and the bacterial cell count obtained by SEM after 96 h in this study. While initial bacterial adhesion was greater than the alkasite, at four days, the biofilm formation was equivalent to the alkasite, due to the possibility of peak fluoride release levels by the glass hybrid restorative up to 96 h. In another study, fluoride releasing GICs showed better biofilm formation inhibition than the resin composite initially but at 96 h the difference between the materials reduced remarkably [32]. Although GICs show sustained low fluoride release for over a month [34], the low concentrations of fluoride released by the GIC may not provide an effective antibacterial action to completely inhibit bacterial adhesion on the surface [35]. Thus, in this study the bacterial counts measured in the SEM images were not significantly different, though the glass hybrid material showed lower counts.

To enhance the physico-mechanical properties of the material, it is advised to combine Equia Forte™ HT Fil with Equia Forte Coat, a light-cured resin coating. Although an improvement in the mechanical and surface smoothness properties has been noted [36], fluoride release is inhibited [33, 36]. An earlier study on the Equia Forte™ HT Fil with and without coating revealed no differences in biofilm formation [37]. In this study, the material was used without the resin coating, however fluoride release could have been inhibited during the first 48 h as the GIC was auto mixed. The fluoride release of auto mixed GICs is lower than that of hand mixed ones because the fluoride remains trapped in the resin matrix [38].

Alkasite is known to release calcium, phosphate and fluoride ions that provide antibacterial properties to the cement particularly against *S. mutans* [8, 39]. Due to the alkaline fillers the material also releases hydroxyl ions which cause acid neutralization of the bacteria [40]. Alkasite exhibits the greatest fluoride release in the first 24 h and a subsequent significant decrease over one week period [41]. The pattern of ion release is like that of GIC, with an initial burst effect followed by sustained slow release by diffusion via cement pores over time [33, 40]. However, the amount of fluoride ions by the alkasite is lower than the GICs over time due to surface modification of fillers which make them resistant to degradation [42]. The study findings for bacterial adhesion measured by OD, CFU and SEM align with the ion release patterns of the alkasite and GICs. Specifically, the alkasite group showed the least bacterial adhesion in the first 24 h as indicated by the OD and CFU values. Despite lower fluoride release by the alkasite, the release of calcium, phosphate and hydroxyl ions with antibacterial effects appears to have potentiated the antibacterial effect of the alkasite. However, the bacterial count on the biofilm after 96-hour incubation of the alkasite was not statistically different from the glass hybrid restorative and nanohybrid groups, as shown by the bacterial cell count and the area covered by the biofilm in the SEM analysis. Thus, bacterial adhesion is determined by properties other than surface roughness, such as ion release patterns of the restorative material that make them bioactive.

Tetric^®^ N-Ceram contains ytterbium-trifluoride (YbF2), which makes it more radiopaque [34]. A steady fluoride release at a low concentration from various composite resins that include YbF2 fillers has been noted. The low fluoride release is attributed to the release of surface-retained fluoride due to the restricted water solubility of YbF2 [5], that resulted in the least inhibition of bacterial adhesion on the resin composite in this study.

SR can influence the bacterial adhesion as rough surfaces provide a greater area for bacterial adhesion. The threshold above which the SR can influence bacterial adhesion is 0.2 μm [21]. All the groups in this investigation had mean SR values less than 0.2 μm. A clinically acceptable range of mean SR values (0.06–0.22 μm) was attained in this study. Therefore, one may conclude that bacterial adhesion was not primarily caused by the range of SR levels in the present study.

When determining the surface smoothness, the filler particle size is a significant factor. The SR will decrease if the filler particle size decreases [23]. Nanohybrid resin composites contain both nano sized and micro sized silica fillers with sizes of 0.02–0.05 μm and 0.3–1 μm respectively [43]. Equia Forte™ HT Fil restorative material is a glass hybrid that has biphasic heterogeneous fillers (normal silica and ultrafine silica fillers). The set cement still contains unreacted glass particles in the polysalt matrix. Therefore, the glass particles are exposed during the finishing and polishing processes when the matrix is abraded [44]. The SR of the glass hybrid restorative material was thus considerably greater in this study than that of the nanohybrid composite.

Comparing alkasites to nanohybid composites, the filler particle sizes of the alkasites are larger, ranging from 0.1 to 35 μm. They also have a filler load of approximately 58% v/v which is lower than that of the nanohybrid resin composites [25, 43]. A previous study revealed that the SR of the alkasite is more than the nanohybrid composite but diminished in comparison with SR values of the GIC [23]. Similar results were observed in this study also, the Ra values of the alkasite were greater than those of the nanohybrid composite. The greater SR of the alkasite in the present study may be due to hand mixing of this material, which raises the possibility of air bubble incorporation [8].

SR and bacterial adhesion on various restorative materials using different polishing techniques have been studied previously [2, 20, 23]. The results show that the SR and bacterial adhesion are dependent on the restorative material and the polishing system type [21]. The correlation between the SR and bacterial adhesion appears to be material-dependent, as conflicting results have been reported in earlier studies [2, 21]. A positive correlation of *S. mutans* adhesion was noted when the SR was above the threshold value 0.2 μm. Within the threshold value, the difference in the SR did not affect the biofilm formation [21]. The Ra values in this study were within the threshold and did not correlate with the bacterial adhesion parameters.

The smoothest surface for any restorative material is obtained using mylar strips during curing following which no finishing and polishing is performed. However, clinical scenarios during restoration require contouring and shaping of the restoration to eliminate high points, which causes roughening of the restoration. To minimise stains on resin composites, the top layer that is rich in resin must be polished [4]. The diamond and aluminium particles of the Sof-Lex ^TM^ polishing system used in this study are harder than the filler particles of the restorative materials leading to smoother surfaces after polishing due to equal removal of the resin matrix and filler particles [45]. An earlier study found that the use of a combination of aluminium oxide and diamond particle two-step polishing system provides better results than the use of only aluminium oxide systems [46], which was hence chosen for this study.

The merits and limitations of this study can be linked to the methodologies used for the measurement of the outcomes. Traditional methods for enumerating bacteria include counting colony-forming units (CFU) using a solid culture medium and measuring the OD using a spectrophotometer [47, 48]. While the CFU method has the advantage of counting only viable bacteria, the OD is a measure of turbidity and thus a proxy measurement of suspended biomass concentration [47]. To determine the CFU the bacterial inoculum was diluted in a ten-fold manner (logarithmic dilutions) plated onto a suitable culture medium and incubated for 1–3 days. The dilution at which the colonies are well separated such that they can be counted by the naked eye in a reproducible manner is determined [47, 48], which was 1:1000 in this study. The accuracy of the method is dependent on the uniform dispersion of *S. mutans* in the biofilm. In addition, clumps of bacterial cells can be counted as single colonies, thus each CFU may consist of one to hundreds of cells [47]. Certain bacteria may remain after vortexing, depending on the surface properties of the material. Additionally, vortexing can destroy bacteria, rendering them unviable for further culturing [49]. The technique is known to be of high sensitivity and specificity but time consuming and labour intensive. Hence this gold standard measure of bacterial adhesion was complemented with other methods of measuring bacterial adhesion, OD measurement and SEM analysis. Both methods have their advantages and disadvantages. OD measurement is a quick and easy method of quantifying bacterial adhesion but is less accurate as the values are influenced by dead and non-viable bacteria, light scattering through the components of the culture media and the sensitivity of the method to bacterial concentrations within a specific range [47]. The use of SEM allows for the description of bacterial morphology and quantification of the bacterial density on surfaces at high resolution. However, dehydration and sputter coating under vacuum can dehydrate and distort the specimens leading to artefacts [50].

In this study, the SR was assessed using a surface profilometer. The average roughness calculated arithmetically, does not reflect the variation in the polished surfaces and fails to provide a full characterization of the SR [46]. While methods like atomic force microscopy can provide more precise values, the profilometer measurements are performed over larger areas giving values that are representative of a wider range of the sample area [4].

Due to the in vitro design, the adhesion of a single bacterial strain in the absence of salivary pellicle attachment was studied, which is another study limitation. The effect of the variety of oral microbial flora is not well reflected. Nevertheless, the single species biofilm model allows for a standardized and reproducible experimental method, and comparison of the results of the studies with similar monospecies microbial models [32]. Additionally, the methods used to determine bacterial adhesion in the present study were not specific for measuring the viability of the biofilm bacteria. The autoclaving of the materials prior to bacterial adhesion experiments may have affected the surface properties and micromorphology of the specimen [51, 52]. Despite the drawbacks of autoclaving, the sterilization was necessitated due to the evidence in literature that the specimens prepared in laboratory can become contaminated with bacteria, either from the material itself or during preparation [51].

Currently, bioactive materials are being used for restoration due to their ability to mineralize residual caries lesions and prevent secondary caries [9]. The study was undertaken with an understanding that the surface characteristics of restorative materials are important, as dental restorative materials are recognized as artificial niches that facilitate the adherence and accumulation of oral microorganisms, leading to oral diseases and affecting the lifespan of restorations. The findings from this in vitro investigation offer insights into the SR and bacterial adhesion of the two newer bioactive restorative materials, glass hybrid restorative and alkasite, compared to a frequently employed material for permanent tooth restoration—a nanohybrid resin composite. These results can assist clinicians in making informed decisions regarding the selection of bioactive restorative materials, based on surface characteristics.

Future in vitro studies should focus on the effect of polymicrobial models on bacterial adhesion to bioactive materials over longer periods of time. Additional clinical research is needed to evaluate the bacterial adhesion of bioactive materials, considering the potential significant influence of bacterial adhesion on restoration prognosis in vivo.

## Conclusion

Within the study limitations, it may be concluded that the alkasite restorative material can inhibit bacterial adhesion to the material better than the glass hybrid restorative and nanohybrid composite. The surface roughness of the nanohybrid composite was less than that of the glass hybrid restorative cement, which did not significantly differ from the alkasite. The surface roughness did not influence the bacterial adhesion to all the three restorative materials. The bacterial adhesion is determined by other properties besides the surface roughness of the material such as ion release patterns for restorative materials with different properties.

## Data Availability

The data that support the findings of this study are available from the corresponding author on reasonable request.

## Abbreviations

SR: Surface roughness
OD: Optical density
OD_650_: Optical density at 650 nm
CFUs: Colony forming units
SEM: Scanning electron microscopy
GIC: Glass ionomer cement
S. mutans: Streptococcus mutans
TNC: Tetric^®^ N-Ceram
EF: Equia forte ^TM^ HT Fil
CN: Cention N^®^
BHI: Brain heart infusion
Ra: Surface roughness value
